# Weight Loss Effects of Once-Weekly Semaglutide 2.4 mg in Adults with and Without Type 2 Diabetes: A Systematic Review and Meta-Analysis

**DOI:** 10.3390/ph18071058

**Published:** 2025-07-18

**Authors:** Boram Hong, Haesoo Kim, Daeun Lee, Kisok Kim

**Affiliations:** 1 College of Pharmacy, Keimyung University, Daegu 42601, Republic of Korea; 2 BK21 Four Center for Forensic Pharmaceutical Sciences, Keimyung University, Daegu 42601, Republic of Korea

**Keywords:** semaglutide, GLP-1 receptor agonist, obesity, type 2 diabetes mellitus, weight loss, meta-analysis

## Abstract

**Background/Objectives**: Semaglutide, a glucagon-like peptide-1 receptor (GLP-1R) agonist, is a well-established pharmacologic agent for inducing weight loss in individuals with obesity and is prescribed regardless of type 2 diabetes mellitus (DM) status. However, it remains unclear whether the weight-lowering efficacy of semaglutide differs significantly between individuals with and without DM. To address this question, we conducted a systematic review and meta-analysis comparing the effects of once-weekly subcutaneous semaglutide at 2.4 mg on weight loss in adults with and without DM. **Methods**: A comprehensive literature search was performed using the PubMed database to identify randomized controlled trials (RCTs) involving overweight or obese adults receiving semaglutide at 2.4 mg weekly for 40 to 70 weeks. Using a random-effects model, we estimated the weighted mean differences in body weight reduction between the two groups. Nine RCTs met the inclusion criteria, among which two provided subgroup data for participants with and without DM within the same trial population. Registration number in PROSPERO: CRD420251077610. **Results**: In participants with DM (n = 4 studies), semaglutide was associated with a weighted mean body weight reduction of −6.34% (95% confidence interval: −6.98 to −5.69), with negligible heterogeneity across studies (I^2^ = 0.0%). By contrast, among participants without DM (n = 7 studies), the weighted estimate of weight loss was −11.57% (95% confidence interval: −12.94 to −10.19), with moderate heterogeneity observed (I^2^ = 63.6%). **Conclusions**: The observed difference in weight loss efficacy between the groups was clinically meaningful. While once-weekly semaglutide at 2.4 mg elicited significant weight loss in both populations, the magnitude of effect was notably greater in those without DM. This disparity may be explained by metabolic characteristics frequently present in individuals with DM, such as insulin resistance, hyperinsulinemia, and compensatory mechanisms related to glycemic control.

## 1. Introduction

Obesity is widely recognized as a chronic, complex, and multifactorial disease [1] that markedly increases the risk of various non-communicable conditions, including type 2 diabetes mellitus (DM) and cardiovascular disease [2]. The World Health Organization defines overweight and obesity as an abnormal or excessive accumulation of adipose tissue that presents a risk to health [3]. For individuals with obesity, weight loss offers multiple clinical benefits, including improved glycemic control, enhanced insulin sensitivity, and reduced cardiovascular risk [4,5].

Among pharmacological options, glucagon-like peptide-1 receptor (GLP-1R) agonists have emerged as a breakthrough in the treatment of obesity [6]. Initially developed for the management of DM, these agents also demonstrate significant efficacy in promoting weight loss—often outperforming other treatments in the same class [7]. Semaglutide, a potent GLP-1R agonist available in both subcutaneous and oral formulations, has shown strong efficacy in appetite suppression, increased satiety, and improvements in various metabolic parameters [8,9]. Extensive research programs—including the Semaglutide Unabated Sustainability in Treatment of Type 2 Diabetes (SUSTAIN) [10], Peptide Innovation for Early Diabetes Treatment (PIONEER) [11], and Semaglutide Treatment Effect in People with Obesity (STEP) [12] trials—have highlighted its superiority in glycemic control and weight reduction compared with other GLP-1R agonists and anti-obesity medications [7,13]. The once-weekly subcutaneous 2.4 mg dose of semaglutide (Wegovy^®^) is specifically approved by the Food and Drug Administration and the European Medicines Agency for chronic weight management in adults with obesity or overweight who have at least one weight-related comorbidity. This approval was based on significant findings across multiple randomized controlled trials (RCTs) [14].

Although semaglutide promotes weight loss in both diabetic and non-diabetic populations, studies have consistently shown a tendency toward reduced weight loss in individuals with DM compared with those without DM [15]. This difference may reflect underlying physiological variations, such as insulin resistance, drug responsiveness, and appetite regulatory mechanisms [16,17]. However, the clinical relevance of this differential response—and whether it is consistent across randomized controlled trials—remains an area requiring further clarification.

Previous meta-analyses, such as Shi et al. (2024) and Kommu and Berg (2024), have assessed semaglutide and GLP-1R agonists for weight loss, but few have stratified outcomes by DM status [7,18]. Therefore, this systematic review and meta-analysis was performed to compare the weight-lowering efficacy of once-weekly subcutaneous semaglutide at 2.4 mg in adults with overweight or obesity, stratified by the presence or absence of DM. By quantifying differences in treatment response based on diabetes status, this analysis aimed to provide evidence to support precision treatment approaches in obesity management.

## 2. Methods

### 2.1. Information Sources and Search Strategy

This systematic review and meta-analysis was conducted in accordance with the PRISMA (Preferred Reporting Items for Systematic Reviews and Meta-Analyses) 2020 guidelines [19]. The protocol was registered in PROSPERO (CRD420251077610). A comprehensive literature search was performed in the PubMed database for studies published up to 2024. The search strategy included the following terms: “semag-lutide”[tiab] AND “2.4 mg” AND “randomized controlled trial” [Publication Type] AND (“obesity” OR “overweight”) AND “weight”.

### 2.2. Eligibility Criteria

We included RCTs that met the following criteria: adult participants with overweight or obesity, an intervention group receiving once-weekly subcutaneous semaglutide at 2.4 mg compared with placebo, treatment duration between 40 and 70 weeks, and percentage change in body weight reported as a primary outcome. Trials with total durations exceeding 70 weeks were eligible if they reported prespecified endpoints (e.g., at 52 or 68 weeks) that fell within the inclusion timeframe. For example, the STEP 5 trial [20], despite its 104-week treatment duration, was included based on the 52-week weight change data, which met our criteria. Data were independently extracted by two reviewers using a standardized data extraction form. The exclusion criteria were overlapping populations across studies, trials limited to participants with prediabetes, lack of data stratified by DM status, and non-RCT formats such as case reports, observational studies, or conference abstracts.

### 2.3. Research Questions

This systematic review and meta-analysis aimed to address the following questions in adults with overweight or obesity:

- What is the efficacy of once-weekly subcutaneous semaglutide at 2.4 mg on body weight reduction in individuals with and without type 2 diabetes mellitus (DM)?
- Is there a difference in weight loss efficacy between individuals with and without DM receiving semaglutide?

### 2.4. Study Selection

**Figure 1:**
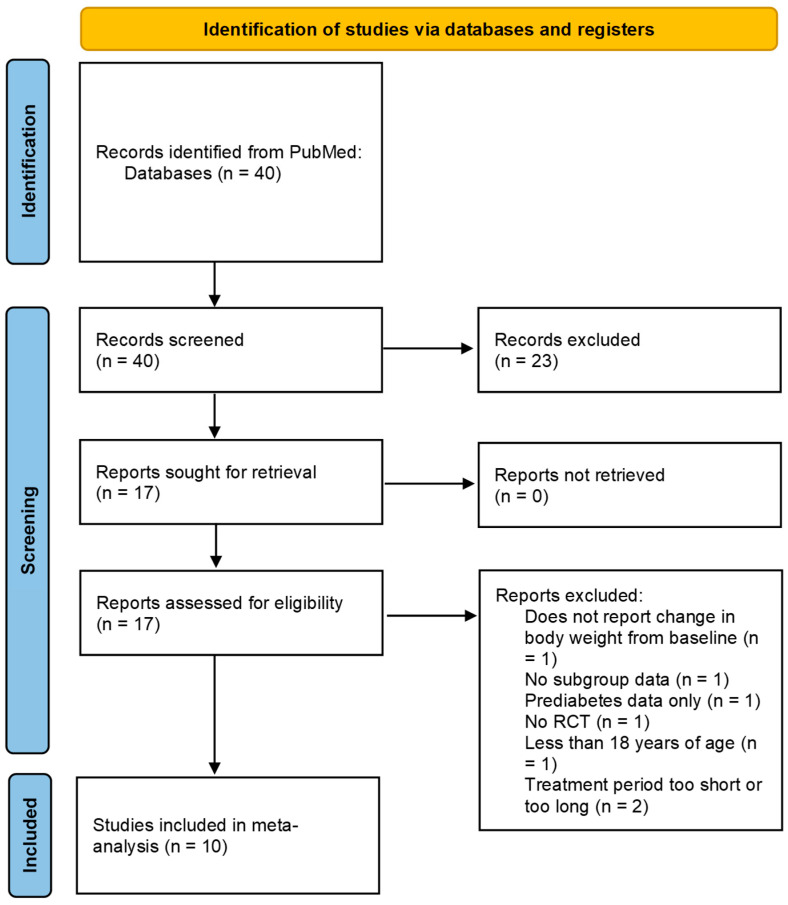
PRISMA flow diagram of study selection for randomized controlled trials of semaglutide use for weight loss in patients with overweight/obesity.

Two reviewers independently screened titles and abstracts using the predefined inclusion criteria. Full texts of potentially eligible studies were retrieved and assessed independently for inclusion. Of 731 full-text articles, 721 were excluded, including studies with overlapping populations (e.g., redundant STEP program datasets), identified by cross-referencing trial registration numbers and participant characteristics. In total, 10 studies were included in the final meta-analysis: STEP 1 [12], STEP 2 [15], STEP 3 [21], STEP 5 [20], STEP 6 [22], STEP 6 post hoc [23], STEP 7 [24], STEP 8 [25], STEP-HFpEF [26], and STEP-HFpEF DM [27]. The study selection process is summarized in Figure 1 (PRISMA flow diagram). STEP 6 and its post hoc analysis were based on the same RCT and used identical datasets. The primary STEP 6 publication was referenced for risk-of-bias assessment and extraction of study design characteristics, whereas the post hoc analysis provided stratified baseline demographic data and subgroup-specific weight change outcomes for participants with and without DM.

### 2.5. Data Extraction

Two reviewers independently extracted data from included studies using a standardized form. Extracted data included study design, location, population characteristics, sample size, treatment arms, treatment duration, primary endpoints, and baseline demographic characteristics (e.g., age, sex, body weight, BMI, waist circumference, and blood pressure). For studies with subgroup data (DM vs. non-DM), outcomes such as percentage change in body weight were extracted.

### 2.6. Outcome Measures

- The efficacy of once-weekly subcutaneous semaglutide at 2.4 mg on the percentage change in body weight in adults with and without DM.
- The difference in weight loss efficacy between individuals with and without DM was assessed as the weighted mean difference (WMD) in percentage body weight change.

Outcomes were reported as percentage change in body weight, with WMDs and 95% confidence intervals (CIs) calculated for DM and non-DM subgroups.

### 2.7. Risk of Bias Assessment

Risk of bias was assessed using the Cochrane Risk of Bias 2.0 tool. Each study was evaluated across five domains: the randomization process, deviations from intended interventions, missing outcome data, outcome measurement, and selection of the reported result. All studies meeting the inclusion criteria were retained for meta-analysis regardless of overall risk-of-bias classification. Assessment of publication bias using funnel plots and Egger’s test was not conducted due to the limited number of included studies.

### 2.8. Data Synthesis

The primary analysis focused on the mean percentage change in body weight in each subgroup (DM vs. non-DM). A random-effects model based on the DerSimonian and Laird method was used to synthesize continuous outcomes, applying inverse variance weighting with the modified Knapp–Hartung method to estimate weighted mean differences (WMDs) and 95% confidence intervals (CIs). Forest plots were generated to visualize individual and pooled effects. Statistical heterogeneity was quantified using the I^2^ and τ^2^ statistics. All meta-analyses were performed using the metafor package in R. A generalized linear model (GLM) was fitted using SAS software (version 9.4; SAS Institute Inc., Cary, NC, USA) to assess whether the weighted mean difference in body weight change between the two groups (DM vs. non-DM) was statistically significant. The results were visualized using a weighted box plot.

## 3. Results

### 3.1. Study Selection

In total, 40 records were identified through the database search, and 17 articles were selected for full-text review after screening titles and abstracts. Of these, seven studies were excluded because of overlapping populations, lack of subgroup data by DM status, or failure to meet the prespecified duration criteria. Consequently, 10 RCTs were included in the meta-analysis (Figure 1).

### 3.2. Study Characteristics

All included trials (Table 1) compared once-weekly subcutaneous semaglutide at 2.4 mg with placebo. Treatment durations ranged from 44 to 68 weeks, with maximum follow-up periods spanning from 51 to 111 weeks. The studies were conducted across diverse geographic regions, including Asia, Europe, North America, and South America, with sample sizes ranging from approximately 300 to more than 1900 participants. Most trials incorporated lifestyle modification interventions, including a hypocaloric diet, increased physical activity, and behavioral counseling. Some studies also included additional dose arms (e.g., 1.0 mg or 1.7 mg) or active comparators, such as liraglutide 3.0 mg.

Baseline demographic characteristics varied among studies, as shown in Table 2 (DM subgroup) and Table 3 (non-DM subgroup). The mean age was 54.8 years in participants with DM and 46.8 years in those without DM. The proportion of female participants was higher in the non-DM group (71.1%) than in the DM group (47.6%). Participants without DM had a higher mean body weight (104.3 vs. 99.1 kg) and body mass index (BMI) (37.5 vs. 35.6 kg/m^2^), while the mean waist circumference was similar between groups (113.6 cm in non-DM vs. 114.1 cm in DM). In some studies, placebo group data were pooled with data from other dose arms or active comparators.

### 3.3. Risk of Bias

**Figure 2:**
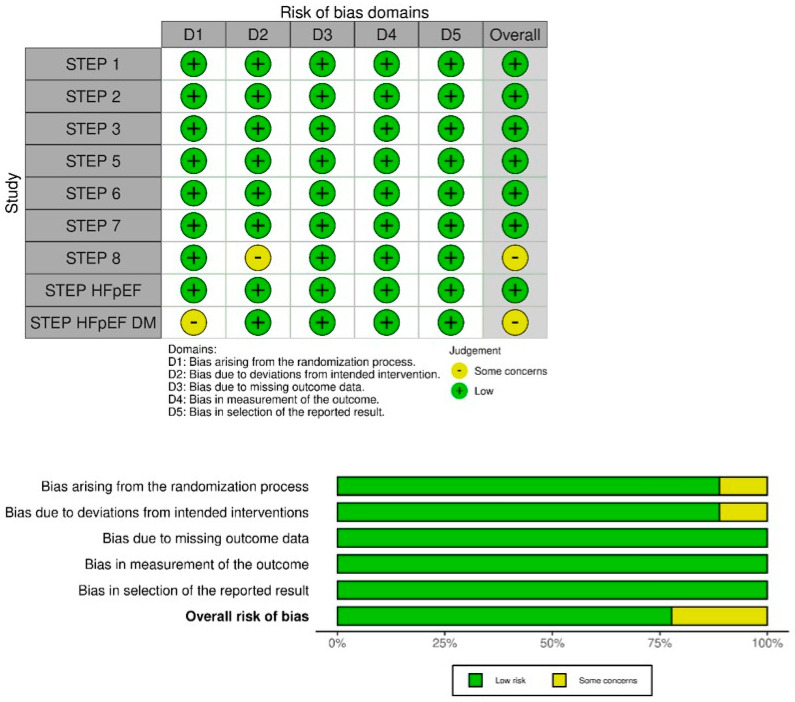
Summary of the Cochrane quality assessment Risk of Bias 2.0 (RoB 2) results stratified by risk domains. The overall risk of bias represents a summary assessment of the potential biases affecting the study’s results.

Risk of bias was assessed for nine studies using the Cochrane Risk of Bias 2.0 tool (Figure 2). Seven studies were judged to have an overall low risk of bias, while two were rated as having some concerns. In the STEP 8 trial, the open-label design raised concerns about potential bias due to deviations from intended interventions. In the STEP-HFpEF DM trial, the method of randomization was not clearly reported, raising concerns about potential bias in the randomization process.

### 3.4. Results of Syntheses

**Figure 3:**
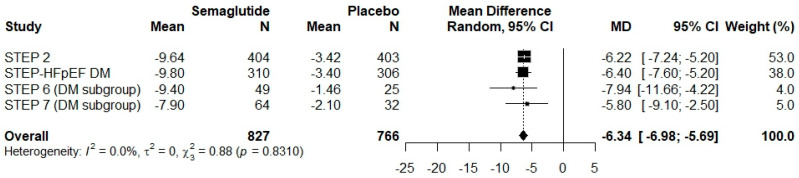
Forest plot showing the mean differences in relative body weight change between semaglutide and placebo in the diabetes (DM) group. The “Overall” row summarizes the pooled mean difference (MD) in body weight change across all included studies.

**Figure 4:**
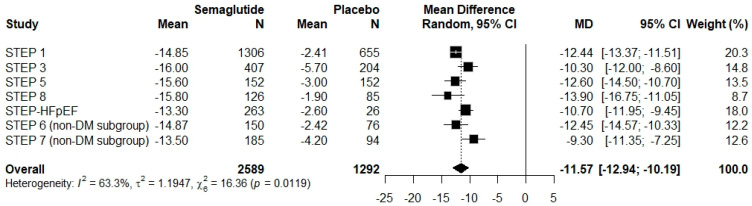
Forest plot showing the mean differences in relative body weight change between semaglutide and placebo in the non-diabetes (non-DM) group. The “Overall” row summarizes the pooled mean difference (MD) in body weight change across all included studies.

**Figure 5:**
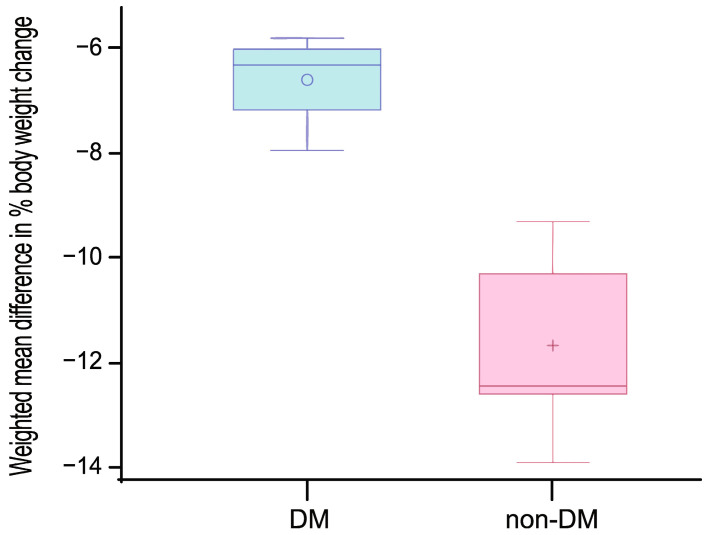
Box plot of the weighted mean difference in body weight change (%) in the semaglutide treatment group compared with the control group, stratified by type 2 diabetes mellitus (DM) status. A statistically significant difference was observed between the DM and non-DM groups (*p* < 0.001).

Meta-analysis of four studies (n = 1872) that reported subgroup data for participants with DM (Figure 3) showed that semaglutide significantly reduced body weight compared with placebo. The WMD was −6.34% (95% CI: −6.98 to −5.69), with negligible heterogeneity (I^2^ = 0%, p = 0.8310), indicating a consistent effect across studies. By contrast, the meta-analysis of seven studies (n = 3842) including participants without DM (Figure 4) demonstrated a more pronounced weight loss effect. The WMD was −11.57% (95% CI: −12.94 to −10.19), with moderate heterogeneity (I^2^ = 63.3%, p = 0.0114), potentially attributable to variations in participant characteristics, intervention protocols, or the timing of weight assessment. Analysis of WMDs using box plots further demonstrated that semaglutide at 2.4 mg induced significantly greater weight loss in individuals without DM than in those with DM (p < 0.001) (Figure 5).

## 4. Discussion

To our knowledge, this is among the first meta-analyses to provide a systematic and quantitative comparison of the weight-lowering efficacy of once-weekly subcutaneous semaglutide at 2.4 mg between individuals with and without DM. Our findings demonstrate that, while semaglutide at 2.4 mg produced clinically meaningful and statistically significant reductions in body weight in both groups compared with placebo, the magnitude of this effect was notably greater among participants without DM (−11.57%) than among those with DM (−6.34%). This ~5% difference is clinically significant, as a 5% weight reduction is associated with improved metabolic and cardiovascular outcomes [4,5]. This observation is consistent with results from individual STEP trials, such as STEP 2 [15], which specifically enrolled patients with DM and reported a mean weight loss of −9.6% with semaglutide at 2.4 mg—a figure that is less than the approximately 15% weight loss observed in the STEP 1 [12] and STEP 3 [21] trials involving participants without DM. The presence of DM appears to attenuate the weight loss response to semaglutide.

Several physiological mechanisms may account for this difference. Individuals with DM typically exhibit insulin resistance and compensatory hyperinsulinemia [28]. GLP-1R agonists, including semaglutide, exert their effects by enhancing glucose-dependent insulin secretion, suppressing glucagon release, delaying gastric emptying, and promoting satiety via central nervous system pathways [6]. These combined actions lead to reduced energy intake and subsequent weight loss. However, in the context of established DM with significant insulin resistance, the incretin effect may be attenuated due to impaired insulin signaling or reduced GLP-1R responsiveness [17]. Furthermore, insulin is an anabolic hormone that promotes fat storage [29]. Chronic hyperinsulinemia, commonly seen in DM, suppresses lipolysis and promotes lipogenesis, which may counteract the weight-reducing effects of semaglutide despite its appetite-suppressing actions [29]. Additionally, improved glycemic control achieved with semaglutide in patients with DM may reduce glycosuria, thereby diminishing caloric loss through urine—a phenomenon sometimes referred to as “glycemic buffering” [30,31]. This ‘glycemic buffering’ effect, where improved glycemic control reduces caloric loss via glycosuria, is supported by studies on GLP-1R agonist responsiveness in DM [30,31,32]. As glycaemia normalizes, this route of caloric loss is reduced, potentially offsetting part of the energy deficit induced by semaglutide and attenuating overall weight loss [31].

Other potential contributors to the differential effect include differences in basal metabolic rate, behavioral factors, and the impact of concomitant medications commonly used in DM management. For instance, some individuals with DM may be taking insulin or sulfonylureas, which are known to be associated with weight gain and could blunt the weight loss achieved with semaglutide [33,34,35]. Additionally, individuals with long-standing DM may have undergone multiple prior dietary and behavioral interventions, potentially reducing the incremental benefit derived from GLP-1R agonist-mediated appetite suppression [36]. On the other hand, combining semaglutide with a high-fiber, low-glycemic diet may enhance weight loss and metabolic control in DM patients by promoting satiety and stabilizing glucose levels, potentially amplifying semaglutide’s effects [4].

Beyond its effects on body weight and glycemic control, semaglutide has demonstrated significant cardiovascular benefits. The SUSTAIN 6 [37] and PIONEER 6 [38] trials reported a reduction in major adverse cardiovascular events in patients with DM at high cardiovascular risk. More recently, the SELECT trial [39], conducted in patients with overweight or obesity and established cardiovascular disease but without DM, also showed a significant reduction in major adverse cardiovascular events with semaglutide at 2.4 mg, alongside substantial weight loss [40]. These findings suggest that the cardiovascular protection offered by semaglutide may extend beyond glucose lowering and weight loss, potentially involving direct effects on atherosclerosis, inflammation, and endothelial function [41,42]. Semaglutide may reduce atherosclerosis by lowering inflammation (e.g., C-reactive protein) and improving endothelial function through GLP-1 receptor–mediated nitric oxide production and reduced vascular oxidative stress [41,42]. These effects may slow plaque progression. Semaglutide has also shown promise in improving renal outcomes in patients with DM and chronic kidney disease, as indicated by the FLOW trial results [43], and in resolving non-alcoholic steatohepatitis without worsening fibrosis in some patients [44].

Despite these benefits, the clinical application of semaglutide is not without challenges. Gastrointestinal adverse events—including nausea, vomiting, diarrhea, and constipation—are the most frequently reported side effects [18,45]. These are generally mild to moderate in severity and tend to be transient, often diminishing over time or with dose-escalation strategies [45]. Importantly, studies suggest that significant weight loss is largely independent of the occurrence of these gastrointestinal side effects [46]. Another critical consideration is the potential for weight regain after discontinuation of treatment. The STEP 1 trial extension [47] and the STEP 4 study [48] showed that a substantial portion of the lost weight was regained within a year of stopping semaglutide, highlighting that obesity is a chronic condition requiring long-term management strategies [49]. This underscores the need for continued research into maintenance therapies, optimal treatment durations, and effective strategies to mitigate weight recidivism [50].

The development of a high-dose (50 mg) oral formulation of semaglutide, as investigated in the OASIS 1 trial [51], has shown weight loss results comparable to those achieved with the subcutaneous 2.4 mg dose. This could offer a more convenient administration route for some patients, provided that long-term efficacy and safety are confirmed [6,51]. The dose-dependent effect of semaglutide on weight loss is well established across various studies, with higher doses generally associated with greater weight reduction [52].

This study has several limitations. First, reliance on post hoc subgroup analyses for DM and non-DM participants in some trials [23,24] may not fully align with the primary study objectives or pre-specified analyses, potentially introducing variability. Second, variations in the intensity of lifestyle interventions (e.g., dietary or behavioral support), treatment duration, and type of behavioral support across trials contributed to heterogeneity, particularly in the non-DM subgroup (I^2^ = 63.3%). Third, pooled placebo data from participants receiving different background therapies or lower-dose arms in some studies [22,25] may introduce bias and influence comparisons. Finally, the limited number of eligible studies precluded sensitivity analyses, subgroup analyses, and publication bias assessments (e.g., funnel plots or Egger’s test). Despite these limitations, this meta-analysis provides a robust quantitative synthesis indicating that the weight-lowering efficacy of semaglutide at 2.4 mg is attenuated in the presence of DM.

Future research should focus on identifying metabolic, genetic, and behavioral predictors of differential treatment responses to semaglutide in both DM and non-DM populations, alongside exploring optimal adjunct therapies, such as dietary or pharmacological interventions, to enhance outcomes and support more personalized obesity management [50]. Understanding the characteristics of “super-responders” versus “non-responders” [50] could help tailor therapeutic choices. For example, female sex has been identified as a potential predictor of hyper-response in some cohorts [53], while the impact of diabetes status itself is a key finding of our study. Additionally, long-term studies are needed to assess the sustainability of weight loss, determine the optimal duration of therapy, and explore strategies to maintain weight loss after semaglutide discontinuation—including the potential role of gradual dose tapering or intermittent therapy.

## 5. Conclusions

This meta-analysis demonstrates that once-weekly subcutaneous semaglutide at 2.4 mg is a highly effective intervention for weight loss in adults with overweight or obesity, both with and without DM. However, the magnitude of weight reduction is significantly greater in individuals without than with DM. This disparity is likely attributable to underlying physiological differences common in DM, such as insulin resistance, chronic hyperinsulinemia, and metabolic adaptations related to improved glycemic control, which may collectively attenuate the weight loss response. These findings underscore the importance of considering an individual’s metabolic status—particularly the presence and characteristics of DM—when selecting and managing pharmacological interventions for obesity. Personalized treatment strategies that account for these metabolic nuances are essential for optimizing the effectiveness of obesity management in diverse patient populations and for fully harnessing the therapeutic potential of agents such as semaglutide.

## Figures and Tables

**Table 1:** Study design and characteristics of randomized controlled trials of once-weekly semaglutide versus placebo for weight loss.

	**STEP-1**	**STEP-2**		**STEP-3**	**STEP-5**
Location	16 countries in Asia, Europe and North and South America	12 countries across Europe, North America, South America, Middle East, South Africa, Asia		United States	Canada, Hungary, Italy, Spain and United States
Population	STEP Program Population *	Adults (age ≥ 18 years) with BMI ≥ 27 kg/m^2^ and ≥1 weight-related comorbidity or BMI ≥ 30 kg/m^2^, with or without type 2 diabetes		STEP Program Population *	STEP Program Population *
Sample Size	1961	1210		611	304
Active Arm	Subcutaneous semaglutide 2.4 mg/week, lifestyle intervention and counseling	Subcutaneous semaglutide 2.4 mg/week, lifestyle intervention and counseling		Subcutaneous semaglutide 2.4 mg/week, lifestyle intervention and counseling	Subcutaneous semaglutide 2.4 mg/week, behavioral intervention
Control	Matching subcutaneous placebo, lifestyle intervention and counseling	Matching subcutaneous placebo, lifestyle intervention and counseling		Matching subcutaneous placebo, lifestyle intervention and counseling	Matching subcutaneous placebo, behavioral intervention
Extra Arm		Subcutaneous semaglutide 1.0 mg/week			
Treatment Period	68 weeks	68 weeks		68 weeks	104 weeks
Primary Endpoint	Percentage change in body weight and weight loss of ≥5% from baseline at week 68	Percentage change in body weight and weight loss of ≥5% from baseline at week 68		Percentage change in body weight and weight loss of ≥5% from baseline at week 68	Percentage change in body weight and weight loss of ≥5% from baseline at week 104
Maximum Follow-up	75 weeks	75 weeks		75 weeks	111 weeks
	**STEP-6**	**STEP-7**	**STEP-8**	**STEP-HFpEF**	**STEP-HFpEF DM**
Location	Japan and South Korea	China, Hong Kong, Brazil, South Korea	United States	13 countries in Asia, Europe and North and South America	16 countries in Asia, Europe and North and South America
Population	Adults (age ≥ 18 years in South Korea, ≥20 years in Japan) with BMI ≥ 27 kg/m^2^ and ≥2 comorbidities, or BMI ≥ 35 kg/m^2^ and ≥1 comorbidity (including hypertension, dyslipidaemia, or type 2 diabetes in Japan)	Adults (age ≥ 18 years) with BMI ≥ 30 kg/m^2^, or BMI ≥ 27 kg/m^2^ with ≥1 weight-related comorbidity (eg, hypertension, dyslipidaemia, OSA, CVD), with or without type 2 diabetes	STEP Program Population *	Persons 18 years of age or older, with a left ventricular ejection fraction of at least 45% and BMI ≥ 30 kg/m^2^	Adults (age ≥ 18 years) with HFpEF (LVEF ≥ 45%), BMI ≥ 30 kg/m^2^, and type 2 diabetes (diagnosed ≥ 90 days before screening, HbA1c ≤ 10%)
Sample Size	401	375	338	529	616
Active Arm	Subcutaneous semaglutide 2.4 mg/week, lifestyle intervention and counseling	Subcutaneous semaglutide 2.4 mg/week, lifestyle intervention and counseling	Subcutaneous semaglutide 2.4 mg/week, lifestyle intervention and counseling	Subcutaneous semaglutide 2.4 mg/week	Subcutaneous semaglutide 2.4 mg/week
Control	Matching subcutaneous placebo, lifestyle intervention and counseling	Matching subcutaneous placebo, lifestyle intervention and counseling	Matching subcutaneous placebo, lifestyle intervention and counseling	Matching subcutaneous placebo	Matching subcutaneous placebo
Extra Arm	Subcutaneous semaglutide 1.7 mg/week, lifestyle intervention and counseling		Subcutaneous liraglutide 3.0 mg/day, lifestyle intervention, and counseling		
Treatment Period	68 weeks	44 weeks	68 weeks	52 weeks	52 weeks
Primary Endpoint	Percentage change in body weight and weight loss of ≥5% from baseline at week 68	Percentage change in body weight and weight loss of ≥5% from baseline at week 44	Percentage change in body weight from baseline at week 68	Change from baseline to week 52 in KCCQ-CSS and percentage change in body weight	Change from baseline to week 52 in KCCQ-CSS and percentage change in body weight
Maximum Follow-up	75 weeks	51 weeks	75 weeks	57 weeks	57 weeks

* Adults (age ≥ 18 years) with BMI ≥ 27 kg/m^2^ with ≥1 weight-related comorbidity or BMI ≥ 30 kg/m^2^, ≥1 self-reported unsuccessful dietary weight loss attempt, and without diabetes.

**Table 2:** Baseline demographic characteristics of participants with diabetes (DM subgroup) enrolled in randomized controlled trials of semaglutide for weight loss.

Study	Sample Size	Age (mean)	Female (%)	Body Weight (kg, mean)	Body Mass Index (kg/m^2^)			Waist Circumference	Blood Pressure (mmHg, mean)	
					Mean	<30 (%)	≥30 (%)		Systolic	Diastolic
**STEP-2**										
Semaglutide	404	55.0	55.2	99.9	35.9	16.8	83.2	114.5	130	80
Placebo	403	55.0	47.1	100.5	35.9	19.1	80.9	115.5	130	80
**STEP-HFpEF DM**										
Semaglutide	310	69.0 (median)	41.3	103.8 (median)	36.9 (median)	0	100	122.0 (median)	132 (median)	79 (median)
Placebo	306	70.0 (median)	47.4	101.7 (median)	36.9 (median)	0	100	118.5 (median)	137 (median)	78 (median)
**STEP-6 (DM Subgroup)**										
Semaglutide	49	54.0	42.9	83.3	31.4	51.0	49.0	102.5		
Placebo *	25	51.0	20.0	95.1	33.3	28.0	72.0	108.1		
**TOTAL** †	1497	54.8	47.6	99.1	35.6	11.8	88.2	114.1	130.0	80.0

Excluding STEP-7 (Does not have subgroup demographic data). * Placebo data for semaglutide 2.4 mg and semaglutide 1.7 mg groups were pooled. † Excluding Step-HFpEF DM median value (age, body weight, body mass index, waist circumference).

**Table 3:** Baseline demographic characteristics of participants without diabetes (non-DM subgroup) enrolled in randomized controlled trials of semaglutide for weight loss.

Study	Sample Size	Age (mean)	Female (%)	Body Weight (kg, mean)	Body Mass Index (kg/m^2^)			Waist Circumference	Blood Pressure (mmHg, mean)	
					Mean	<30 (%)	≥30 (%)		Systolic	Diastolic
**STEP-1**										
Semaglutide	1306	46.0	73.1	105.4	37.8	6.2	93.8	114.6	126	80
Placebo	655	47.0	76.0	105.2	38.0	5.5	94.5	114.8	127	80
**STEP-3**										
Semaglutide	407	46.0	77.4	106.9	38.1	5.7	94.3	113.6	124	80
Placebo	204	46.0	88.2	103.7	37.8	7.4	92.6	111.8	124	81
**STEP-5**										
Semaglutide	152	47.3	80.9	105.6	38.6			115.8	126	80
Placebo *	152	47.4	74.3	106.5	38.5			115.7	125	80
**STEP-8**										
Semaglutide	126	48.0	81.0	102.5	37.0	7.1	92.9	111.8	125	81
Placebo ‡	85	51.0	77.6	108.8	38.8	4.7	95.3	115.4	123	79
**STEP-HFpEF**										
Semaglutide	263	70.0 (median)	56.7	104.7 (median)	37.2 (median)	0	100	119.0 (median)	133.0 (median)	78.0 (median)
Placebo	266	69.0 (median)	55.6	105.3 (median)	36.9 (median)	0	100	120.0 (median)	132.0 (median)	78.0 (median)
**STEP-6 (Non-DM Subgroup)**										
Semaglutide	150	51.0	42.7	88.1	32.3			104.2		
Placebo *	76	49.0	27.6	88.6	31.5			102.4		
**TOTAL** †	3842	46.8	71.1	104.3	37.5	5.1	94.9	113.6	125.6	80.1

Excluding STEP-7 (Does not have subgroup demographic data). * Placebo data for semaglutide 2.4 mg and semaglutide 1.7 mg groups were pooled. ‡ Placebo data for semaglutide and liraglutide groups were pooled. † Excluding Step-HFpEF DM median value (age, body weight, body mass index, waist circumference).

## Data Availability

No new data were created or analyzed in this study. Data sharing is not applicable to this article.
